# Reduced Toxicological Activity of Cigarette Smoke by the Addition of Ammonia Magnesium Phosphate to the Paper of an Electrically Heated Cigarette: Subchronic Inhalation Toxicology

**DOI:** 10.1080/08958370701813273

**Published:** 2008-05-05

**Authors:** O. Moennikes, P. M. Vanscheeuwijck, B. Friedrichs, E. Anskeit, G. J. Patskan

**Affiliations:** Philip Morris Products S.A., PMI Research and Development, Neuchâtel, Switzerland; Philip Morris Research Laboratories bvba, Leuven, Belgium; Philip Morris Research Laboratories GmbH, Cologne, Germany; Philip Morris USA, Richmond, Virginia, USA

## Abstract

Cigarette smoke is a complex chemical mixture that causes a variety of diseases, such as lung cancer. With the electrically heated cigarette smoking system (EHCSS), temperatures are applied to the tobacco below those found in conventional cigarettes, resulting in less combustion, reduced yields of some smoke constituents, and decreased activity in some standard toxicological tests. The first generation of electrically heated cigarettes (EHC) also resulted in increased formaldehyde yields; therefore, a second generation of EHC was developed with ammonium magnesium phosphate (AMP) in the cigarette paper in part to address this increase. The toxicological activity of mainstream smoke from these two generations of EHC and of a conventional reference cigarette was investigated in two studies in rats: a standard 90-day inhalation toxicity study and a 35-day inhalation study focusing on lung inflammation. Many of the typical smoke exposure-related changes were found to be less pronounced after exposure to smoke from the second-generation EHC with AMP than to smoke from the first-generation EHC or the conventional reference cigarette, when compared on a particulate matter or nicotine basis. Differences between the EHC without AMP and the conventional reference cigarette were not as prominent. Overall, AMP incorporated in the EHC cigarette paper reduced the inhalation toxicity of the EHCSS more than expected based on the observed reduction in aldehyde yields.

Cigarette smoke is a complex mixture that causes a variety of diseases, such as lung cancer, chronic obstructive pulmonary disease (COPD), and cardiovascular disease (U.S. Department of Health and Human Services, 2004; International Agency for Research on Cancer, 2004). It has been hypothesized that the chemical composition of mainstream smoke (MS) and consequently its toxicological effects depend to a great extent on the burning temperature of the tobacco (Patskan & Reininghaus, 2003). Supportive evidence stems from pyrolysis experiments with tobacco showing a decreased yield of many toxic MS constituents with decreasing temperature (Torikai et al., 2004). The mutagenicity of cigarette smoke condensate was also found to be reduced when tobacco was pyrolyzed at temperatures below those found in conventional cigarettes (White et al., 2001). One approach using the association between lower temperature and reduced toxicological effects is the electrically heated cigarette smoking system (EHCSS), which comprises an electronically controlled heating device in a specially designed lighter as well as specially designed cigarettes. These electrically heated cigarettes (EHC) are inserted into the smoking device, which allows precise regulation of the time course and amount of energy supplied to the tobacco during each puff by means of heater blades (Patskan & Reininghaus, 2003). This controlled heating of the tobacco results in a distinctly lower temperature applied to the tobacco than that found in the burning cone of conventional cigarettes. Because of its design, the EHCSS produces practically no sidestream smoke between puffs, and because of the lower temperature, the composition of MS from the EHCSS differs considerably from that of conventional cigarettes (Stabbert et al., 2003).

With the first-generation EHCSS, in vitro experiments showed lower toxicological responses compared to conventional reference cigarettes for cytotoxicity in the neutral red assay and genotoxicity of total particulate matter (TPM) in the *Salmonella* reverse mutation assay and the mouse lymphoma thymidine kinase assay (Tewes et al., 2003; Roemer et al., 2004; Schramke et al., 2006). In vivo results from a 90-day inhalation study in rats were not quite as conclusive as in vitro results (Terpstra et al., 2003): When the effects of MS from the EHCSS were compared to those of a conventional reference cigarette, the University of Kentucky Reference Cigarette 1R4F (Diana & Vaught, 1990), results indicated the overall toxicity of diluted MS from the EHCSS to be lower than that of the 1R4F when calculated on a per cigarette basis, but not when calculated on the basis of equal TPM concentration in the exposure chambers (Terpstra et al., 2003). This can be attributed mainly to the lower TPM yield per cigarette for the EHCSS compared to the 1R4F.

Apart from significant reductions in the yields of almost all analyzed toxicologically relevant constituents in the MS of the first-generation EHCSS compared to that from the conventional reference cigarette, the formaldehyde concentration per milligram TPM was approximately sevenfold higher (Stabbert et al., 2003). Because formaldehyde is a low-temperature degradation product, this increase was attributed to the reduction in maximum temperature from ∼950°C for a conventional cigarette to ∼600°C for the EHCSS (Patskan & Reininghaus, 2003). One approach used to mitigate this was to attempt to influence the trapping of formaldehyde by other MS constituents. For the second-generation EHCSS, this concept was realized by replacing CaCO_3_ in the cigarette paper with ammonium magnesium phosphate (AMP) (Fournier & Paine, 2001). The intention was to decrease formaldehyde by chemical reaction with ammonia released from AMP, bearing in mind that this might yield condensation products, such as hexamethylenetetramine (HMT, also called urotropin). The impact of AMP incorporation on the chemical composition of MS and with in vitro test systems is reported and discussed in detail in a parallel publication (Roemer et al., 2008). Briefly, AMP incorporation resulted in decreased concentrations of a variety of toxic MS constituents, including formaldehyde, with decreased cytotoxicity in the neutral red uptake assay and decreased TPM mutagenicity in the *Salmonella* reverse mutation assay. To complement the valuable information on toxicity obtained from the chemical analysis and these in vitro tests, in vivo tests were performed. The objective of the current study was to investigate the toxicological activity in rats of MS from two generations of EHC (with and without AMP in the cigarette paper) and a conventional reference cigarette, the 1R4F. Two studies were conducted: a standard 90-day inhalation toxicity study with endpoints chosen according to OECD guideline 413, and a 35-day inhalation study focusing on lung inflammation. This extension of the standard inhalation toxicity assay with a pulmonary inflammation assay was designed to address the growing interest in the role of altered inflammatory processes in smoke-related diseases (U.S. Department of Health and Human Services, 2004).

## MATERIALS AND METHODS

### Experimental Design

Two subchronic cigarette smoke inhalation studies in rats were conducted: a 90-day inhalation toxicity study and a 35-day pulmonary inflammation study.

#### 90-Day Inhalation Toxicity Study

Guideline 413 from the Organization for Economic Cooperation and Development (OECD) guidelines for the testing of chemicals (Organization for Economic Cooperation and Development, 1981) has been adapted for the evaluation of cigarette modifications (e.g., Ayres et al., 2001; Vanscheeuwijck et al., 2002; Terpstra et al., 2003; Higuchi et al., 2004; Carmines & Gaworski, 2005). The inhalation toxicity of MS from a first-generation EHC (EHC-CaCO_3_) and a second-generation EHC with AMP in the cigarette paper (EHC-AMP) was compared to that of a conventional reference cigarette, the University of Kentucky Reference Cigarette 1R4F. MS from the 1R4F was diluted to three TPM target concentrations, i.e., 80 μg/L (low), 120 μg/L (medium), and 160 μg/L (high), to allow for the establishment of a concentration-response curve. This concentration-response curve was used for the evaluation of the effects of EHCSS MS (target concentration: 90 μg TPM/L). The MS exposure concentration for the two EHCs was chosen based on the inhalation toxicity determined with the first-generation EHCSS (Terpstra et al., 2003) so that most of the anticipated biological effects would be in the same range as those of the 1R4F. Each exposure group was made up of 10 male rats and 10 female rats; control groups were exposed to filtered, conditioned fresh air (sham). The reversibility and/or progression of MS-related effects were investigated at the end of a 42-day postexposure period. For this purpose, satellite groups of 10 male and 10 female rats each for sham, the EHC-CaCO_3_, the EHC-AMP, and the highest dose level of the 1R4F were kept without treatment for an additional 42 days after the end of the exposure period.

#### 35-Day Pulmonary Inflammation Study

Pulmonary inflammation was determined by differentiation of subsets of free lung cells in bronchoalveolar lavage fluid (BALF). Inflammation response induced by MS from the EHC-AMP (750 μg TPM/L) was compared to that of the 1R4F, which was diluted to 450 μg TPM/L and 600 μg TPM/L to allow determination of a concentration-response curve. The 35-day inhalation period is sufficient for detecting changes in the distribution of free lung cells in bronchoalveolar lavage fluid (Friedrichs et al., 2006). Each exposure group (including sham) was made up of 12 female rats. No significant differences in response for female and male rats have been reported.

### Animals

Five-week-old outbred Sprague Dawley rats (Crl:CDBR), bred under specific-pathogen-free conditions, were obtained from Charles River Germany. The rats were barrier-maintained in an animal laboratory unit with controlled hygienic conditions. The laboratory air (filtered fresh air) was conditioned, and positive pressure was maintained in the animal area. For the 90-day inhalation toxicity study, room temperature was maintained at 21 ± 1°C and relative humidity at 55 ± *5%.* The light/dark cycle was 12 h/12 h. Environmental conditions in the inflammation study were similar and also in the comfort zone for the rats. Good hygienic conditions were maintained as evidenced by the low number of colony-forming units on the microbiological media used for screening of the diet and drinking water, as well as of the laboratory air and surfaces. Prestudy pathological and histopathological examination of the respiratory tract, as well as serological screening (Terpstra et al., 2003), revealed that the rats were suitable for use in inhalation studies.

Identification, housing, feeding, and watering were performed as previously described (Vanscheeuwijck et al., 2002). Chemical analytical screening revealed the diet to be in conformity with NTP specifications (National Toxicology Program, 1992). After an acclimatization period of 7 days, rats were allocated systematically to the exposure groups to achieve the same body weight in all groups.

### Cigarette Smoke Exposure

#### Cigarettes

The Reference Cigarette 1R4F was obtained from the Tobacco and Health Institute at the University of Kentucky (Diana & Vaught, 1990). In the absence of an existing cigarette that closely matches the EHCSS, the 1R4F was selected because it is a low “tar” American-blend cigarette, which is representative of the current sales-weighted U.S. cigarette market (Roemer et al., 2004). In addition, it is readily available to the general scientific community and can therefore serve as a reference cigarette in the product testing of novel cigarettes (Coggins et al., 1989). The first-generation EHCSS is described in detail elsewhere (Patskan & Reininghaus, 2003) and on the Internet at www.pmusa-science.com. Briefly, the EHCSS consists of two parts, an electronically controlled smoking device (lighter/heater) and specially designed cigarettes (EHCs). Apart from some changes in the heater design, the main difference between the first- and second-generation EHCSS is a change in the composition of the cigarette paper: The second generation has AMP incorporated in the cigarette paper. Due to ongoing technical development, there are also some minor differences in the physical design and temperature program of the heater device used in the inflammation study compared to that used in the toxicity study. These minor differences are not considered to affect the outcome of the study.

#### Smoke Generation

The cigarettes were conditioned according to ISO standard 3402 (International Organization for Standardization, 1991a) and smoked in basic conformity with ISO standards 3308 (International Organization for Standardization, 1991b) and 4387 (International Organization for Standardization, 1991c). Some minor deviations from the ISO standards were necessary for technical reasons (Stabbert et al., 2003).

MS from the 1R4F was generated on 30-port smoking machines (type SM85, Philip Morris Research Laboratories GmbH, Germany; Reininghaus & Hackenberg, 1977), each equipped with a 4-piston pump (Baumgartner and Coggins, 1980). The MS was diluted with filtered, conditioned fresh air to produce the desired 1R4F concentrations and conveyed via glass tubing to the exposure chamber. In both studies, the puff count ranged from 8.2 ± 0.5 to 8.9 ± 0.5 puffs/cigarette and the cigarettes were smoked to an average butt length of 34.3 ± 1.4 to 35.1 ± 1.2 mm. Puff volumes were 35.0 ml on average with amaximum SD of 0.4 ml.

**FIG. 1:**
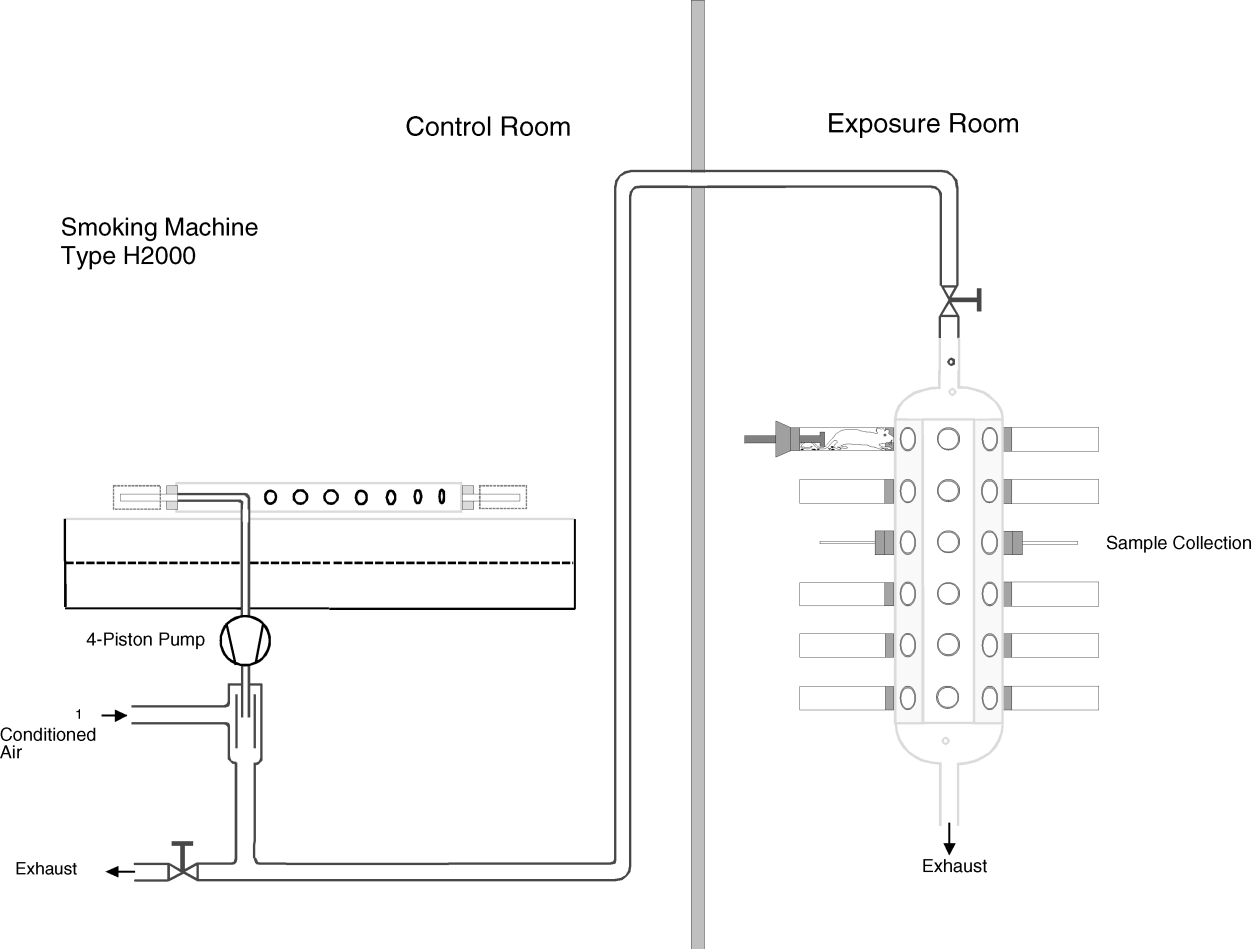
Mainstream smoke generation and exposure for the EHC. Smoking machine, tubing and exposure chamber.

MS from the EHCSS was generated as previously described (Stabbert et al., 2003). Briefly, modified 30-port PMRL smoking machines (type H2000 and H-IFL, Philip Morris Research Laboratories GmbH, Germany) each equipped with a 4-piston pump and 30 electric heaters (Figure 1) were used for smoke generation. The total average heating energy per puff was approximately 24 J, applied during a 2-s puff (Patskan & Reininghaus, 2003). The puff count for the EHCSS is fixed to 8 puffs by design. Puff volumes were 35.1 ml on average with amaximum SD of 0.4 ml.

#### Exposure

For the 90-day inhalation toxicity study, the flow rate through the exposure chambers was between 92 and 96 L/min, and the mean age of the diluted MS was 11s. The mean temperature of the test atmospheres in the exposure chambers was 23 ± 2°C. The relative humidity of the filtered, conditioned fresh air used for dilution and sham exposure was 60 ± 6%. Environmental conditions in the inflammation study were similar and also in the comfort zone for the rats.

The exposure regimen in the inhalation toxicity study was 6 h per day, 7 days per week for 90 days with a 42-day postexposure period. The exposure regimen in the inflammation study was 2 × 1 h per day (with a 30-min break between the 2 hours), 7 days per week for 35 days with no postexposure period.

To monitor the stability and reproducibility of the smoke generation, TPM, CO, nicotine, aldehydes, and particle size distribution in the exposure chambers (at the breathing zone of the rats, Figure 1) were determined according to previously described analytical methods (Haussmann et al., 1998a).

### Biological Endpoints

All rats were checked daily for mortality, moribundity, signs of toxicity, and injury. Three systematically selected rats per sex and group were examined specifically for general condition and behavior shortly after each daily exposure. Body weight (individual) and food consumption (group-wise, inhalation toxicity study) were determined at least once per week.

Respiratory parameters measured by head-out single-chamber plethysmography, steady-state proportion of blood carboxyhemoglobin (COHb) (Klimisch et al., 1974; Tyuma et al., 1981), and representative nicotine metabolites in 24-h urine were determined in the inhalation toxicity study as previously described (Rustemeier et al., 1993; Haussmann et al., 1998b). Full necropsy (inhalation toxicity study) was performed without prior fasting on the day following the last day of exposure or post exposure, respectively (Vanscheeuwijck et al., 2002). The weights of the lungs with larynx and trachea and of the liver, brain, adrenal glands, testes, kidneys, thymus, and spleen were determined. Hematological and clinical chemistry parameters were determined according to standard methods. Histological sectioning and staining of all OECD-required organs (Organization for Economic Cooperation and Development, 1981) with special emphasis on the respiratory-tract organs was performed as described (Terpstra et al., 2003). All slides were evaluated in a blind manner by a veterinary pathologist and scored according to a defined grading system (0 = no finding; 1 = slight; 2 = slight/moderate; 3 = moderate; 4 = moderate/marked; 5 = marked) (Teredesai et al., 2000).

#### Analysis of Pulmonary Inflammation

Lavage and flow cy-tometric differentiation of BALF cells was performed as previously described (Friedrichs et al., 2006). Viability of BALF cells for all groups was 96% to 97% (SE: 0.3–0.5%); i.e., cell viability was not affected by smoke inhalation. Cells were stained with the FITC-labeled anti-rat granulocyte monoclonal mouse antibody, clone HIS48 (BD Biosciences).

### Evaluation and Statistics

Arithmetic means and measures of variance were calculated as descriptive statistics using textbook formulas. Particle size distribution was calculated by linear regression analysis after probit transformation (Finney, 1971) of the cumulative frequencies and logarithmic transformation of the aerodynamic diameter values.

To determine 1R4F MS-related effects, the smoke-exposed groups were compared with the sham-exposed groups. In the case of a significant result, this overall comparison was followed by a pairwise comparison between the sham-exposed group and each of the smoke-exposed groups. For continuous data, analysis of variance or the Kruskal–Wallis *H*-test (if at least 1 data point is below the quantification limit) were applied for overall comparison followed by Dunnett's test (Dunnett, 1955). For ordinal data, the Cochran–Mantel–Haenszel test (Koch & Edwards, 1988) was applied for both overall and pairwise comparisons. To determine EHCSS MS-related effects, the smoke-exposed groups were compared with the sham-exposed group using the *t*-test for continuous data and the Cochran–Mantel–Haenszel test for ordinal data. In all tests, *p* values less than or equal to 0.05 were considered to be significant (two-tailed). No adjustments for multiple testing were made. Statistically significant results should be considered as explorative indicators rather than confirmatory evidence.

**FIG. 3:**
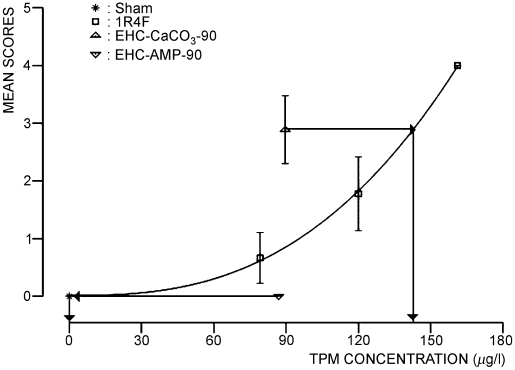
EECR estimation for atrophy of olfactory epithelium in the olfactory region of the nose at level 2. Male rats at the end of the inhalation period of the subchronic inhalation toxicity study. Data points with error bars (mean values with SE) represent the scoring of histopathological findings for the different exposure groups. For the 1R4F cigarette the concentration response curve is plotted on a μg TPM/L basis. Arrows indicate the estimation of the concentration of 1R4F MS causing an effect comparable to the one of the EHC MS. The EECR is calculated by dividing the corresponding 1R4F concentration through the EHC concentration.

To assess differences between each EHCSS and the 1R4F, equal effect concentration ratios (EECRs) were determined (Terpstra et al., 2003) for individual endpoints, if the concentration-response curve for the 1R4F was significantly different from zero, the mean response of at least one EHCSS group was significantly different from sham, and the mean response lay between the mean response for sham and the highest 1R4F group; however, extrapolation of the 1R4F dose-response curves was made when necessary. To find the EECRs, concentration-response curves were calculated for the sham and 1R4F groups with least-squares fit to the power function *y* = a*x^b^* + *c.* Interpolation of the effects of the EHCSS to equal effects on the 1R4F concentration-response curve indicates the concentration of MS from the 1R4F needed to produce an equal effect (for example, see Figure 3). The ratio of these two concentrations is defined as the EECR, which is reciprocal to the relative toxicological activity of the two cigarette types. An EECR of less than 1 means that the toxicological activity of the EHCSS is lower than that of the 1R4F, and an EECR of more than 1 means that the toxicological activity of the EHCSS is higher than that of the 1R4F. EECRs assume qualitatively similar concentration-response curves for all MS types. They are point estimates, for which no measure of variability can be determined, but still provide an overview indication of relative toxicological potency. EECRs can be calculated on the basis of each MS constituent for which analytical data are available.

## RESULTS

### Inhalation Toxicity Study: Composition of Diluted Mainstream Smoke

There was little variation in the composition of the test atmospheres for each specific test sample per condition throughout the conduct of the study, and TPM concentrations were in good compliance with target concentrations (Table 1). The particle size distribution measurements indicated that particles were equally respirable in all smoke-exposed groups (Table 1). Of the individual MS constituents determined, only nicotine and acrolein concentrations in MS from the two EHCs were between those of 1R4F-low and 1R4F-high. CO was lower for both EHCs compared to 1R4F-low (−84% for EHC-AMP and −70% for EHC-CaCO_3_). Formaldehyde was within the range of the 1R4F for the EHC-AMP (comparable to 1R4F-medium), but 6 times higher for the EHC-CaCO_3_ compared to 1R4F-high. Acetaldehyde was lowest for the EHC-AMP (−45% compared to EHC-CaCO_3_ and −27% compared to 1R4F-low). The low CO concentration and relatively high formaldehyde concentration in the EHC-CaCO_3_ test atmosphere (compared to the 1R4F) have been seen before in inhalation and analytical studies (Stabbert et al., 2003; Terpstra et al., 2003) . The reduction in acetaldehyde and formaldehyde concentrations in MS from the EHC-AMP to a level comparable to that of 1R4F-medium is the result of incorporation of AMP in the second-generation EHC (Roemer et al., 2008). In addition, the use of AMP in the cigarette paper resulted in a further 49% decrease in CO concentration.

**TABLE 1:** Characterization of test atmospheres*: inhalation toxicity study

Parameter	Sham	1R4F-low	1R4F-medium	1R4F-high	EHC-CaCO_3_	EHC-AMP
TPM (μg/l)	<0.9	79 ± 4	120 ± 5	161 ± 8	90 ± 5	87 ± 5
Particle size, MMAD (μm)	—	0.46	0.46	0.47	0.39	0.44
GSD	—	1.65	1.66	1.66	1.71	1.78
Carbon monoxide (ppm)	<1.5	94 ± 5	141 ± 8	188 ± 9	28.7 ± 0.8	14.5 ± 0.7
Nicotine (μg/l)	<0.03	5.9 ± 0.4	8.6 ± 0.6	11.2 ± 1.1	8.3 ± 0.7	7.3 ± 0.6
Formaldehyde (ppm)	—	0.12 ± 0.02	0.17 ± 0.03	0.22 ± 0.03	1.32 ± 0.10	0.16 ± 0.03
Acetaldehyde (ppm)	—	4.0 ± 0.3	5.9 ± 0.4	7.6 ± 0.6	5.3 ± 0.1	2.9 ± 0.2
Acrolein (ppm)	—	0.27 ± 0.03	0.40 ± 0.04	0.52 ± 0.03	0.42 ± 0.03	0.35 ± 0.03

* Measured at breathing zone in the exposure chambers.

Values represent means ± standard deviations.

MMAD: mass-median aerodynamic diameter; GSD: geometric standard deviation.

### Inhalation Toxicity Study: Biomonitoring

Respiratory parameters, steady-state blood carboxyhemoglobin concentration, and nicotine metabolites in urine were determined and used as biomarkers of exposure (Table 2).

**TABLE 2:** Biomonitoring: inhalation toxicity study

Parameter	Sex	Sham	1R4F-low	1R4F-medium	1R4F-high	EHC-CaCO_3_	EHC-AMP
Respiratory frequency (1/min)	M	129 ± 8	152 ± 8*	128 ± 5	121 ± 4	131 ± 9	125 ± 10
	F	119 ±10	146 ± 10	111 ± 7	96 ± 6	100 ± 4	141 ± 5
Average inspiratory flow (ml/sec)	M	8.7 ± 0.4	6.2 ± 0.4*	5.4 ± 0.6*	5.9 ± 0.4*	6.0 ± 0.4*	6.7 ± 0.6*
	F	7.1 ± 0.6	5.7 ± 0.6	4.7 ± 0.5*	3.9 ± 0.6*	4.7 ± 0.4*	6.3 ± 0.4
COHb (%)	M	0.64 ± 0.03	13.94 ± 0.30*	19.5 ± 0.53*	26.00 ± 0.65*	4.65 ± 0.29*	2.27 ± 0.07*
	F	0.85 ± 0.03	12.82 ± 0.33*	20.38 ± 1.21*	25.27 ± 0.57*	4.91 ± 0.40*	1.52 ± 0.06*
Recovery of inhaled nicotine (%)	M	— (n.d.)	73.2 ± 5.3	61.5 ± 6.8	76.0 ± 7.3	63.7 ± 5.7	60.4 ± 7.3
	F	— (n.d.)	68.1 ± 9.1	88.3 ± 9.4	75.5 ± 3.9	68.4 ± 11.4	69.6 ± 4.7

Values represent means ± standard error.

* Statistically significantly different from sham, p ≤ 0.05.

n.d.: not determined.

#### Respiratory Physiology

Respiratory depression in rodent inhalation studies is a reaction to respiratory-tract irritation (Alarie, 1973) and is usually concentration dependent in cigarette smoke inhalation studies (Terpstra et al., 2003). Average inspiratory flow (AIF) was lower in the 1R4F groups compared to sham (Table 2). AIF in the EHC-CaCO_3_ groups was comparable to that in the 1R4F-low group (males) and the 1R4F-medium group (females). AIF in the EHC-AMP group was in the range between sham and the low 1R4F group, indicating a lower effect of MS from the EHC-AMP on respiration than MS from the other cigarettes in the study. While the respiratory frequency was reduced in the groups exposed to 1R4F MS in a concentration-dependent manner (both sexes, not statistically significant) and the results for the groups exposed to EHC-AMP or EHC-CaCO_3_ MS fit into this concentration-response range, the relevance of this result is difficult to interpret because the results of the sham-exposed group did not fit to the typical concentration-response curve seen in previous studies (Terpstra et al., 2003).

#### Carboxyhemoglobin

Steady-state concentrations of carboxyhemoglobin (COHb) were approximately 5% in the EHC-CaCO_3_ group, approximately 2% in the EHC-AMP group, and up to 26% in the 1R4F high group, thus corresponding to the CO concentration in the test atmosphere. The results for the 1R4F are in agreement with historical mainstream smoke inhalation data from our laboratory (data not shown).

#### Urinary Nicotine Metabolites

The amount of nicotine metabolites excreted in urine correlates well with the estimate of nicotine inhaled (calculated from the respiratory minute volume [Guyton, 1947], the daily exposure duration, and the nicotine concentration in diluted smoke). The excretion of these metabolites (60% to 88%, Table 2) was in the range of or higher than the 60% expected (Schepers et al., 1993). The higher excretion may suggest that nicotine was taken up not only by inhalation, but also via other routes, such as oral ingestion during grooming (Haussmann et al., 1998b).

In summary, respiratory physiology determinations, COHb-levels in blood, and urinary nicotine metabolite profiles indicate that the rats inhaled MS doses corresponding to the concentrations of the representative MS constituents in the test atmospheres.

### Inhalation Toxicity Study: Biological Results

#### Body Weight

**FIG. 2:**
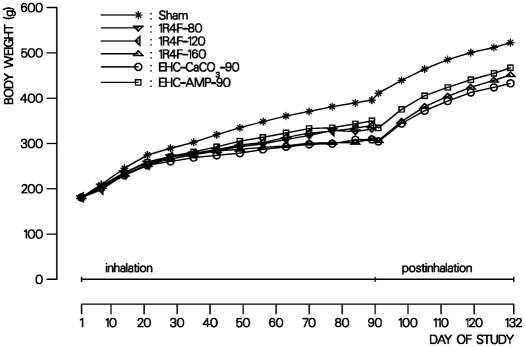
Body weight development of male rats in the subchronic inhalation toxicity study. Curves represent the mean body weight of sham- and MS-exposed groups.

Body weight of all rats increased throughout the study. At 90 days, body weight was significantly lower for all male smoke-exposed rats compared to sham (from −12% in the EHC-AMP group to −22% in the EHC-CaCO_3_ group) (Figure 2), while females were unaffected (data not shown). At the end of the 42-day postexposure period, body weight was still significantly lower for male smoke-exposed rats (−11% to −17% compared to sham, Figure 2). This is due to an unusually high body weight in male sham-exposed rats. Smoke exposure-related body weight depression in males and the lack of such an effect in females has often reported in smoke inhalation studies of comparable design with Sprague-Dawley rats (Coggins et al., 1989; Vanscheeuwijck et al., 2002; Terpstra et al., 2003). It is considered a general systemic effect of toxicity. Smoke constituents, such as nicotine (Chowdhury et al., 1989; Chowdhury, 1990) and acrolein (Bouley et al., 1975; Feron et al., 1978), have been implicated in this effect. Food consumption normally correlates with body weight development. The mean food consumption relative to body weight was, thus, similar for all groups and ranged from 8.0 ± 1.0 to 8.8 ±1.2 g/(100-g rat × day) for male rats and from 9.3 ± 1.3 to 10.8 ± 1.0 g/(100-g rat × day) for female rats.

EECRs were obtained by comparing the mean body weight gain reduction in male rats at the end of the 90-day inhalation period for each of the EHCSS groups to the fitted concentration-response curve of the 1R4F. EECRs for the reduction in body weight gain were 2.0 for the EHC-CaCO_3_ and 0.6 for the EHC-AMP (Table 3), suggesting less pronounced body weight effects by exposure to MS from the EHC-AMP and more pronounced body weight effects by exposure to MS from the EHC-CaCO_3_ compared to exposure to MS from the1R4F, when compared on a TPM basis.

**TABLE 3:** Body weight and hematology after 90 days of exposure: inhalation toxicity study

		Groups						EECRs	
			
Parameter	Sex	Sham	1R4F-Iow	lR4F-medium	lR4F-high	EHC-CaCO_3_	EHC-AMP	EHC-CaCO_3_	EHC-AMP
Body weight (g)	M	395.8 ± 6.7	331.7 ± 11.8*	339.7 ± 9.9*	310.3 ± 10.1*	309.0 ± 7.7*	349.9 ± 10.8*	1.99	0.61
	F	212.4 ± 3.9	199.4 ± 3.8	204.5 ± 4.7	207.7 ± 5.2	202.1 ± 4.4	200.8 ± 5.9	–	–
Platelet count (10^9^/l)	M	977 ± 42	934 ± 43	817 ± 49*	862 ± 32	851 ± 29	818 ± 38	1.62	2.26
	F	921 ± 58	901 ± 38	886 ± 37	812 ± 23	880 ± 36	906 ± 26	–	–
Neutrophils in blood (10^9^/l)	M	0.87 ± 0.16	1.14 ± 0.19	1.48 ± 0.20	1.90 ± 0.30*	1.39 ± 0.13	1.34 ± 0.36	1.24	1.21
	F	0.45 ± 0.05	0.74 ± 0.10	1.09 ± 0.14*	1.30 ± 0.13*	1.04 ± 0.10*	0.64 ± 0.08*	1.34	0.61
Lymphocytes in blood (10^9^/l)	M	6.32 ± 0.54	3.74 ± 0.64*	3.83 ± 0.54*	3.91 ± 0.41*	5.23 ± 0.56	4.82 ± 0.82	–	–
	F	3.61 ± 0.45	3.33 ± 0.30	3.56 ± 0.42	3.75 ± 0.48	2.67 ± 0.22	3.79 ± 0.55	–	–

Values represent means ± standard error.

* Statistically significantly different from sham, p ≤ 0.05.

EECRs were calculated on an equal TPM basis.

–: no EECR calculated because data did not fit the criteria.

Typical smoke exposure-related findings were observed during the inhalation period. These included Harderian gland secretion and effects on spontaneous activity in a few rats, although there was no difference between groups.

#### Clinical Chemistry

In the 1R4F groups, concentration-dependent and statistically significant changes compared to sham were seen for a few parameters in female rats at 90 days; the most prominent was an increase in alkaline phosphatase (AP) activity of +96% (Table 4). Most of the effects reversed during the postexposure period (data not shown).

**TABLE 4:** Clinical chemistry after 90 days of exposure: inhalation toxicity study

		Groups						EECRs	
			
Parameter	Sex	Sham	1R4F-Iow	lR4F-medium	lR4F-high	EHC-CaCO_3_	EHC-AMP	EHC-CaCO_3_	EHC-AMP
Total cholesterol (mmol/1)	M	2.0 ± 0.1	1.5 ± 0.1*	1.5 ± 0.1*	1.6 ± 0.1*	1.7 ± 0.1	1.8 ± 0.1	–	–
	F	2.3 ± 0.2	1.6 ± 0.2*	1.5 ± 0.1*	1.1 ± 0.1*	1.2 ± 0.1*	1.8 ± 0.1*	1.75	0.68
Triglycerides (mmol/1)	M	0.8 ± 0.1	0.7 ± 0.1	0.7 ± 0.1	0.9 ± 0.1	0.8 ± 0.1	0.9 ± 0.1	–	–
	F	1.1 ± 0.3	0.7 ± 0.1	0.5 ± 0.1*	0.4 ± 0.0*	0.5 ± 0.0*	0.8 ± 0.2	1.25	0.62
ALT (UA)	M	55.2 ± 12.9	50.8 ± 3.2	54.3 ± 4.6	63.1 ± 9.1	43.4 ± 2.5	43.9 ± 4.0	–	–
	F	32.1 ± 1.8	34.6 ± 1.8	48.0 ± 4.2*	54.6 ± 3.0*	45.6 ± 3.1*	44.2 ± 5.2	1.36	1.33
AP(UA)	M	193.4 ± 16.0	217.2 ± 12.5	214.5 ± 17.6	275.9 ± 36.5	260.4 ± 35.8	212.5 ± 19.2	–	–
	F	108.8 ± 6.9	158.3 ± 11.0	197.4 ± 23.2*	213.7 ± 14.6*	219.9 ± 19.1*	132.2 ± 11.0	1.84	0.38
Protein (g/1)	M	61.4 ± 0.8	57.7 ± 1.3*	59.2 ± 1.0	61.3 ± 1.0 59.9 ± 1.3	59.9 ± 1.3	62.4 ± 1.1	–	–
	F	66.9 ± 1.9	61.6 ± 1.5*	60.1 ± 1.1*	55.7 ±1.1*	56.7 ± 0.5	61.9 ± 2.6	1.71	0.98

Values represent means ± standard error.

* Statistically significantly different from sham, p ≤ 0.05.

EECRs were calculated on an equal TPM basis.

–: no EECR calculated because data did not fit the criteria.

AP: alkaline phosphatase activity.

ALT: alanine aminotransferase activity.

A comparison of the EHCSS groups with sham revealed no statistically significant differences for male rats, and only a few for female rats. Of the four statistically significant differences seen for the EHC-CaCO_3_ group, the most prominent was for AP activity (+102%). The single statistically significant difference seen in the EHC-AMP group was a slight decrease in total cholesterol concentration (−22%). Most effects had reversed by the end of the postexposure period (data not shown). In general, these effects lacked any correlation to pathological findings in major organs, such as the liver or kidney, and were thus not considered to be toxicologically relevant or to adversely affect the health status of the rats. Comparable changes have been observed in other smoke inhalation studies (Vanscheeuwijck et al., 2002; Terpstra et al., 2003) and are thought to reflect differences in the nutritional status of smoke-exposed rats and the influence of smoke exposure on lipid metabolism (Latha et al., 1988; Maida & Howlett, 1990).

Mean EECRs for clinical parameters for female rats were 1.6 for the EHC-CaCO_3_ and 0.8 for the EHC-AMP (Table 4). These EECRs may indicate on average a slightly lower impact by exposure to MS from the EHC-AMP than by exposure to MS from the EHC-CaCO_3_ or the 1R4F, when compared on a TPM basis.

#### Hematology

Red blood cell (RBC) parameters (data not shown) and platelet counts for all groups were within the range of values for untreated rats given by the breeder, while a trend towards lower platelet counts was seen in smoke-exposed groups (Table 3). The total white blood cell (WBC) count was lower in male smoke-exposed rats compared to sham (data not shown), which is in accordance with our previous MS inhalation studies (Vanscheeuwijck et al., 2002). In females, the WBC count was slightly higher in all smoke-exposed groups compared to sham; however, some of the sham values obtained were unusually low compared to those given by the breeder for untreated rats (data not shown). Histopathological examination of the bone-marrow (data not shown) did not reveal any changes that could explain this lower total WBC count in these rats.

With regard to the differential WBC count, a concentration-dependent increase in segmented neutrophils and decrease in lymphocytes was observed (Table 3) in the smoke-exposed groups (not statistically significant in all groups). This is similar to findings in previous MS inhalation studies in our laboratories (Vanscheeuwijck et al., 2002; Terpstra et al., 2003), but was not seen by others (Coggins et al., 1989; Gaworski et al., 1997). Exposure to cigarette smoke has been reported to promote the release of neutrophils from bone marrow into the peripheral blood (Terashima et al., 1997, 1999) and in humans is associated with increased neutrophilic chemotactic activity (Anderson et al., 1991). EECRs for hematological parameters were 1.4 (males) and 1.3 (females) for the EHC-CaCO_3_ and 1.7 (males) and 0.6 (females) for the EHC-AMP, which is an inconclusive response due to the large variation of the EECRs.

At the end of the 42-day postexposure period, no significant differences were seen between the sham and smoke-exposed groups for RBC parameters, total WBC count, and differential WBC count, indicating complete recovery (exception: slight increase in platelet count in female rats exposed to MS from the EHC-CaCO_3_, which is considered a chance finding; data not shown).

#### Gross Pathology

Typical smoke-related findings, such as discoloration of the fur and thymus atrophy (Vanscheeuwijck et al., 2002), were seen, along with a variety of incidental findings (data not shown). Only discoloration of the fur was still seen at the end of the postexposure period.

#### Organ Weights

Relative organ weights (data not shown) were higher at 90 days for lungs (up to +38%, males only), adrenals (up to +66%), and kidneys (up to +19%, males only), and lower for thymus (up to −58%). These changes are in accordance with published data (Huber et al., 1981; Coggins et al., 1989; Vanscheeuwijck et al., 2002; Terpstra et al., 2003) and are considered to be caused by smoke-related irritation and exposure-related stress. The increase in adrenal weight might well be an effect of nicotine (Boelsterli et al., 1984). Almost all organ weight changes had recovered completely by the end of the 42-day postexposure period, although thymus atrophy data were inconsistent. Mean EECRs for relative organ weights were 1.8 (males) and 1.6 (females) for the EHC-CaCO_3_ and 0.4 (males) and 0 (females) for the EHC-AMP, suggesting lower toxicological impact by exposure to MS from the EHC-AMP and slightly higher toxicological impact by exposure to MS from the EHC-CaCO_3_ compared to that of the 1R4F, when compared on a TPM basis.

#### Histopathology of Respiratory-Tract Organs (Table 5)

In the nose, the histopathological changes observed in all smoke-exposed groups included reserve-cell hyperplasia of the respiratory epithelium on the lateral wall, the nasoturbinates, and the maxilloturbinates; goblet-cell hyperplasia and squamous metaplasia accompanied by the loss of goblet cells in the septal region of the anterior nose section (level 1, immediately posterior to the incisor teeth); reserve-cell hyperplasia of the respiratory epithelium and atrophy (Figure 3) and squamous metaplasia of the olfactory epithelium at nose level 2 (at incisive papilla); and atrophy and squamous metaplasia of the olfactory epithelium at nose level 3 (at the second palatal ridge) and nose level 4 (at the first upper molar teeth). Mean EECRs for the nose were 1.4 (males) and 1.5 (females) for the EHC-CaCO_3_ and 0.2 (males) and 0.4 (females) for the EHC-AMP, indicative of lower nasal irritant effects from exposure to MS from the EHC-AMP and slightly higher irritative effects from exposure to MS from the EHC-CaCO_3_ compared to that of 1R4F, when compared on a TPM basis.

**TABLE 5:** Histopathology of respiratory tract organs after 90 days of exposure: inhalation toxicity study Scores of histopathological findings and laryngeal epithelial thickness

		Groups						EECRs	
			
Parameter	Sex	Sham	1R4F-low	1R4F-medium	1R4F-high	EHC-CaCO_3_	EHC-AMP	EHC-CaCO_3_	EHC-AMP
Nose, level 1									
Reserve cell hyperplasia (respiratory epithelium)	M	0.0 ± 0.0	4.0 ± 0.0*	4.0 ± 0.0*	4.0 ± 0.0*	4.0 ± 0.0*	3.1 ± 0.4*	–	–
	F	0.0 ± 0.0	2.2 ± 0.2*	2.6 ± 0.3*	3.6 ± 0.3*	2.4 ± 0.3*	2.0 ± 0.0*	1.07	0.85
Goblet cell hyperplasia (respiratory epithelium)	M	0.0 ± 0.0	0.6 ± 0.2*	1.3 ± 0.2*	1.5 ± 0.2*	0.2 ± 0.1	0.4 ± 0.2*	0.34	0.62
	F	0.0 ± 0.0	0.4 ± 0.2*	0.4 ± 0.2*	0.7 ± 0.2*	0.1 ± 0.1	0.1 ± 0.1	–	–
Loss of goblet cells (respiratory epithelium)	M	0.0 ± 0.0	0.7 ± 0.2*	1.6 ± 0.2*	1.5 ± 0.2*	1.9 ± 0.2*	0.1 ± 0.1	–	–
	F	0.0 ± 0.0	0.7 ± 0.2*	1.6 ± 0.3*	1.8 ± 0.1*	1.3 ± 0.2*	0.2 ± 0.1	–	–
Squamous metaplasia (respiratory epithelium)	M	0.0 ± 0.0	1.3 ± 0.5*	3.1 ± 0.1*	2.5 ± 0.4*	2.9 ± 0.1*	0.7 ± 0.4	1.85	0.22
	F	0.4 ± 0.4	0.3 ± 0.3	2.3 ± 0.5*	1.8 ± 0.6*	1.1 ± 0.4	0.0 ± 0.0	–	–
Nose, level 2									
Reserve cell hyperplasia (respiratory epithelium)	M	0.0 ± 0.0	0.0 ± 0.0	0.9 ± 0.5	1.7 ± 0.3*	1.1 ± 0.5*	0.0 ± 0.0	1.48	0.43
	F	0.0 ± 0.0	0.0 ± 0.0	0.0 ± 0.0	0.3 ± 0.3	0.0 ± 0.0	0.0 ± 0.0	–	–
Atrophy (olfactory epithelium)	M	0.0 ± 0.0	0.7 ± 0.4	1.8 ± 0.6*	4.0 ± 0.0*	2.9 ± 0.6*	0.0 ± 0.0	1.6	0
	F	0.0 ± 0.0	0.2 ± 0.2	2.9 ± 0.5*	3.7 ± 0.3*	3.2 ± 0.3*	0.3 ± 0.2	1.62	0.58
Squamous metaplasia (olfactory epithelium)	M	0.0 ± 0.0	0.0 ± 0.0	1.0 ± 0.5	2.1 ± 0.5*	0.7 ± 0.4	0.0 ± 0.0	–	–
	F	0.0 ± 0.0	0.1 ± 0.1	1.4 ± 0.4*	1.3 ± 0.2*	1.1 ± 0.1*	0.0 ± 0.0	1.47	0.22
Nose, level 3									
Atrophy (olfactory epithelium)	M	0.0 ± 0.0	0.4 ± 0.4	1.6 ± 0.6*	4.0 ± 0.0*	2.2 ± 0.7*	0.0 ± 0.0	1.49	0.36
	F	0.0 ± 0.0	0.4 ± 0.4	3.0 ± 0.4*	3.8 ± 0.2*	3.2 ± 0.3*	0.0 ± 0.0	1.59	0.26
Squamous metaplasia (olfactory epithelium)	M	0.0 ± 0.0	0.3 ± 0.3	1.0 ± 0.5	3.0 ± 0.0*	2.0 ± 0.5*	0.0 ± 0.0	1.61	0
	F	0.0 ± 0.0	0.2 ± 0.2	1.3 ± 0.3*	2.0 ± 0.2*	1.2 ± 0.2*	0.0 ± 0.0	1.39	0.32
Nose, level 4									
Atrophy (olfactory epithelium)	M	0.0 ± 0.0	0.2 ± 0.2	0.7 ± 0.3	2.0 ± 0.0*	1.1 ± 0.4*	0.0 ± 0.0	1.52	s0
	F	0.0 ± 0.0	0.1 ± 0.1	1.6 ± 0.2*	1.9 ± 0.1*	1.6 ± 0.2*	0.0 ± 0.0	1.57	0.31
Squamous metaplasia (olfactory epithelium)	M	0.0 ± 0.0	0.3 ± 0.3	1.0 ± 0.5	3.0 ± 0.0*	1.7 ± 0.5*	0.0 ± 0.0	1.52	0
	F	0.0 ± 0.0	0.1 ± 0.1	1.4 ± 0.3*	1.6 ± 0.2*	1.5 ± 0.4*	0.0 ± 0.0	1.68	0.28
Larynx, base of epiglottis	M	0.0 ± 0.0	4.7 ± 0.0*	5.0 ± 0.0*	5.0 ± 0.0*	5.0 ± 0.0*	5.0 ± 0.0*	–	–
Squamous metaplasia (pseudostratified epithelium)	F	0.0 ± 0.0	5.0 ± 0.0*	5.0 ± 0.0*	5.0 ± 0.0*	5.0 ± 0.0*	5.0 ± 0.0*	–	–
Hyperplasia (Squamous epithelium)	M	0.0 ± 0.0	5.0 ± 0.0*	5.0 ± 0.0*	5.0 ± 0.0*	5.0 ± 0.0*	5.0 ± 0.0*	–	–
	F	0.0 ± 0.0	5.0 ± 0.0*	5.0 ± 0.0*	5.0 ± 0.0*	5.0 ± 0.0*	5.0 ± 0.0*	–	–
Arytenoid projections, ventral depression squamous metaplasia (cuboidal epithelium)	M	0.0 ± 0.0	0.9 ± 0.3*	1.1 ± 0.4*	2.3 ± 0.6*	0.9 ± 0.4*	0.9 ± 0.3*	1.03	1.06
	F	0.0 ± 0.0	0.7 ± 0.4	2.4 ± 0.6*	3.2 ± 0.6*	0.7 ± 0.4	1.2 ± 0.4*	0.73	1.02
Arytenoid projections, vocal cords, lower medial region hyperplasia (squamous epithelium)	M	0.0 ± 0.0	2.3 ± 0.2*	2.9 ± 0.2*	2.4 ± 0.2*	2.3 ± 0.2*	2.8 ± 0.2*	–	–
	F	0.0 ± 0.0	3.0 ± 0.3*	2.3 ± 0.3*	2.8 ± 0.3*	2.0 ± 0.3*	3.2 ± 0.2*	–	–
Arytenoid projections, vocal cords, upper medial region	M	0.0 ± 0.0	2.0 ± 0.4*	2.6 ± 0.7*	2.9 ± 0.4*	1.0 ± 0.2*	2.1 ± 0.4*	0.23	0.99
Squamous metaplasia (pseudostratified epithelium)	F	0.0 ± 0.0	4.6 ± 0.2*	3.3 ± 0.4*	3.4 ± 0.3*	3.3 ± 0.7*	3.3 ± 0.6*	–	–
Arytenoid projections, floor of the larynx	M	0.0 ± 0.0	4.6 ± 0.3*	5.0 ± 0.0*	4.8 ± 0.2*	4.8 ± 0.2*	4.8 ± 0.2*	–	–
Squamous metaplasia (pseudostratified epithelium)	F	0.0 ± 0.0	4.2 ± 0.6*	4.8 ± 0.2*	5.0 ± 0.0*	5.0 ± 0.0*	4.8 ± 0.2*	–	–
Arytenoid projections, vocal folds	M	0.0 ± 0.0	0.8 ± 0.2*	1.4 ± 0.2*	1.5 ± 0.2*	0.7 ± 0.3*	1.2 ± 0.3*	0.62	1.35
Hyperplasia (squamous epithelium)	F	0.0 ± 0.0	1.2 ± 0.2*	1.0 ± 0.1*	1.1 ± 0.1*	0.9 ± 0.1	1.2 ± 0.2*	–	–
Tracheal bifurcation									
reserve cell hyperplasia (respiratory epithelium)	M	0.0 ± 0.0	0.0 ± 0.0	0.0 ± 0.0	0.1 ± 0.1	0.0 ± 0.0	0.0 ± 0.0	–	–
goblet cell hyperplasia (respiratory epithelium)	M	0.0 ± 0.0	0.3 ± 0.2	0.4 ± 0.3	0.9 ± 0.4	0.3 ± 0.2	0.1 ± 0.1	–	–
goblet cell hyperplasia (respiratory epithelium)	F	0.2 ± 0.1	0.3 ± 0.2	0.9 ± 0.3	0.4 ± 0.3	0.4 ± 0.2	0.2 ± 0.1	–	–
Left lung									
Goblet cell hyperplasia (respiratory epithelium)	M	0.6 ± 0.6	2.9 ± 0.5*	3.0 ± 0.5*	3.3 ± 0.5*	3.0 ± 0.5*	0.9 ± 0.4	1.16	0
	F	0.4 ± 0.3	1.2 ± 0.6	2.3 ± 0.6*	3.2 ± 0.6*	2.6 ± 0.7*	1.2 ± 0.6	1.53	0.83
Alveolar macrophages (respiratory epithelium)	M	0.0 ± 0.0	0.8 ± 0.1*	1.0 ± 0.2*	1.3 ± 0.2*	0.3 ± 0.2	0.1 ± 0.1	–	–
	F	0.0 ± 0.0	0.3 ± 0.2	0.7 ± 0.2*	1.3 ± 0.2*	0.1 ± 0.1	0.0 ± 0.0	–	–
Larynx									
ventral depression (μm)	M	7.73 ± 0.45	11.16 ± 0.68	11.66 ± 0.70*	14.72 ± 1.74*	11.61 ± 0.98*	11.85 ± 0.78*	1.14	1.23
	F	7.95 ± 0.47	10.56 ± 0.71	15.74 ± 1.63*	16.54 ± 1.01*	11.22 ± 0.78*	11.88 ± 1.09*	0.80	0.95
floor of the larynx (μm)	M	9.56 ± 0.34	17.51 ± 1.24*	24.74 ± 0.53*	21.67 ± 2.37*	20.64 ± 1.74*	22.73 ± 1.57*	1.17	1.72
	F	8.62 ± 0.11	18.97 ± 1.18*	23.52 ± 1.51*	29.29 ± 1.61*	20.44 ± 1.29*	24.68 ± 1.64*	1.04	1.45
vocal cords (μm)	M	18.66 ± 1.11	29.39 ± 1.28*	34.39 ± 1.38*	32.21 ± 0.89*	29.51 ± 1.55*	32.65 ± 1.22*	–	–
	F	17.42 ± 1.55	33.16 ± 0.94*	32.33 ± 1.22*	34.03 ± 1.02*	31.93 ± 1.25*	34.91 ± 1.44*	–	–

Values represent means ± standard error.

* Statistically significantly different from sham, p ≥ 0.05.

EECRs were calculated on an equal TPM basis.

–: no EECR calculated because data did not fit the criteria.

In the larynx, hyperplasia of the squamous epithelium was observed in smoke-exposed rats ventromedially at the base of the epiglottis, at the vocal cords lower medial region, and at the vocal folds. Additional findings included hyperplasia and squamous metaplasia of the cuboidal epithelium at the ventral depression; hyperplasia of the pseudostratified epithelium at the vocal cords (upper medial region); and squamous metaplasia of the pseudostratified epithelium ventrolaterally, at the base of the epiglottis, at the floor of the larynx, and at the vocal cords upper medial region. Morphometrical determinations showed increased epithelial thickness at the ventral depression, floor of the larynx, and vocal cords. Mean EECRs for the larynx were 0.8 (males) and 0.9 (females) for the EHC-CaCO_3_ and 1.3 (males) and 1.1 (females) for the EHC-AMP. These EECRs, calculated on a TPM basis, in concordance with the morphologic and morphometric data, suggest minimal differences between the effects of the MS from the two generations of EHC and that of the 1R4F, although with an indication of a slightly higher toxicological impact for MS from the EHC-AMP compared to the EHC-CaCO_3_ and the 1R4F.

In the trachea, minimal reserve-cell hyperplasia and goblet-cell hyperplasia were observed in smoke-exposed rats. The left lung showed goblet-cell hyperplasia of the bronchial epithelium and accumulation of pigmented alveolar macrophages in the alveolar lumen. EECRs for the lung were 1.2 (males) and 1.5 (females) for EHC-CaCO_3_ and 0 (males) and 0.8 (females) for the EHC-AMP, again indicative of lower toxicological potency for MS from the EHC-AMP and slightly higher potency for MS from the EHC-CaCO_3_ MS compared to that of the 1R4F, when compared on a TPM basis.

At the end of the 42-day postexposure period, the goblet-cell hyperplasia in the anterior nose was slightly higher than at the end of the 90-day exposure period. This has been observed in earlier studies and has also been reported in the literature (Harkema et al., 1989; Hotchkiss et al., 1995). Most other findings reversed completely or nearly completely, except for reserve-cell hyperplasia and squamous metaplasia of the respiratory epithelium in the anterior nose (1R4F and EHC-CaCO_3_), atrophy of the olfactory epithelium in the posterior nose (1R4F and EHC-CaCO_3_), and hyperplasia of the squamous epithelium at the vocal cords (all groups). This is consistent with recovery effects seen in previous studies, suggesting that the MS exposure-related morphological changes are predominantly adaptive, reversible responses to repeated irritation (Burger et al., 1989).

#### Histopathology of Non-Respiratory-Tract Organs

No histopathological effect was seen in non-respiratory-tract organs and tissues, apart from a minimal to moderate involution/atrophy of the thymus in smoke-exposed groups, as indicated by the lower thymus weight. Laboratory animal thymus atrophy via CD4+CD8+ lymphocyte apoptosis has been associated with any type of stress, such as restraint, irritation, trauma, or social stress, which is mediated by glucocorticoid release from the adrenal cortex (Warholm et al., 1984; Nakanishi et al., 1998; Dal-Zotto et al., 2003; Rodrigues-Mascarenhas et al., 2006).

### Pulmonary Inflammation Study

The pulmonary inflammation study was designed to complement the inhalation toxicity study with lung inflammation data. A limited number of quality control parameters were determined, e.g., for biomonitoring.

#### Composition of Diluted Mainstream Smoke

The MS composition for the EHC-AMP and 1R4F in this inflammation study (Table 6) corresponded well to that observed in the inhalation toxicity study (Table 1).

**TABLE 6:** Characterization of test atmospheres*: pulmonary inflammation study

Parameter	Sham	1R4F-450^a^	1R4F-600^a^	EHC-AMP-750^a^
TPM (μg/l)	<4.0	448 ± 30	603 ± 41	739 ± 38
Carbon monoxide (ppm)	<1.5	505 ± 34	656 ± 47	74.1 ± 5.4
Nicotine (μg/l)	<0.11	31 ± 2.0	41 ± 3.1	65.1 ± 3.1
Formaldehyde (ppm)	–	0.58 ± 0.04	0.75 ± 0.05	0.83 ± 0.17
Acetaldehyde (ppm)	–	20 ± 0.6	27 ± 20	18 ± 1.2
Acrolein	–	1.5 ± 0.1	1.9 ± 0.1	2.0 ± 0.10

* Measured at breathing zone in the exposure chambers.

Values represent means ± standard deviations.

a Target TPM concentration (μg/l).

#### Biomonitoring

The decrease in respiratory frequency and the increase in carboxyhemoglobin concentration showed a clear concentration response (data not shown). As in the inhalation toxicity study, the biomonitoring parameters indicated that the rats inhaled increasing doses with increasing smoke concentrations in the test atmospheres.

#### Inflammation

**FIG. 4:**
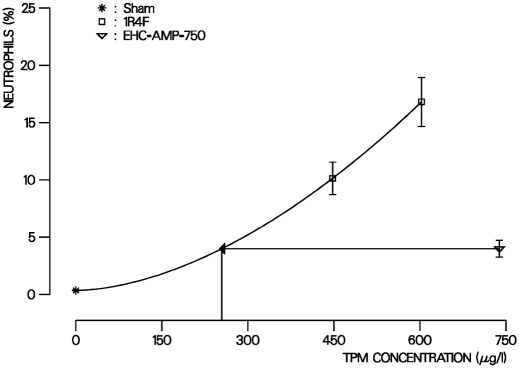
EECR estimation for the relative number of BALF neutrophils in the inflammation study. Data points with error bars (mean values with SE of indicated exposure groups) represent percentage of neutrophils in free lung cells determined after 35 days of exposure. For the 1R4F the concentration response curve is plotted on a μg TPM/L basis. Arrows indicated the estimation of the concentration of 1R4F MS causing an effect comparable to the one of the EHC MS. The EECR is calculated by dividing the corresponding 1R4F concentration through the EHC concentration.

A statistically significant increase in the absolute and relative number of neutrophils in BALF (a main indicator of pulmonary inflammation) was found in all smoke-exposed groups compared to sham in the pulmonary inflammation study. Similar responses have been seen in other cigarette smoke inhalation studies in rodents (Gairola, 1986; Mordelet-Dambrine et al., 1991; Balansky et al., 1992) and have been attributed to the inhalation of the particulate phase of MS in these models (Friedrichs et al., 2006). The increase in neutrophils showed a clear concentration-response relationship in the 1R4F groups (Table 7 and Figure 4). The number of neutrophils was considerably lower in the EHC-AMP group compared to the 1R4F groups (Table 7 and Figure 4).

**TABLE 7:** Differentiation of BALF cells after 90 days of exposure: pulmonary inflammation study

	Groups				
		
Parameter	Sham	1R4F-450	1R4F-600	AMP-750	EHC-AMP-750
FLC number(10^6^)	12.6 ± 0.8	16.7 ± 0.7*	17.0 ± 0.8*	15.6 ± 0.6	–
Alveolar macrophages (10^6^)	12.5 ± 0.7	14.9 ± 0.7	14.0 ± 0.9	14.9 ± 0.6*	–
Alveolar macrophages (%)	99.0 ± 0.1	89.2 ± 1.4*	82.1 ± 2.2*	95.5 ± 0.8*	–
Neutrophils (10^6^)	0.04 ± 0.008	1.70 ± 0.283*	2.76 ± 0.301*	0.63 ± 0.118*	0.33
Neutrophils (%)	0.35 ± 0.05	10.13 ± 1.42*	16.80 ± 2.14*	3.99 ± 0.73*	0.35
Lymphocytes (10^6^)	0.08 ± 0.012	0.11 ± 0.007	0.18 ± 0.02*	0.078 ± 0.007	–
L	0.65 ± 0.10	0.67 ± 0.05	1.10 ± 0.15*	0.50 ± 0.05	–

Values represent means ± standard error.

* Statistically significantly different from sham, p ≤ 0.05.

EECRs calculated on an equal TPM basis.

–: no EECR calculated.

Other exposure-related changes in BALF were increases in the absolute number of alveolar macrophages, the absolute and relative number of lymphocytes, and the total number of free bronchoalveolar cells, and a decrease in the relative number of alveolar macrophages. The concentration-response effect for these parameters was relatively weak compared to the neutrophils, as observed in other MS inhalation studies (Friedrichs et al., 2006). Therefore, only neutrophils were used for EECR calculations, i.e., 0.3 and 0.4 for absolute and relative number of neutrophils, respectively, which suggests that MS from the EHC-AMP has approximately one-third the inflammatory potential of MS from the 1R4F.

### DISCUSSION

One strategy to reduce the adverse biological effects of MS is to reduce the yield of certain toxic smoke constituents by generating smoke at temperatures below those found in conventional cigarettes (Patskan & Reininghaus, 2003). The EHCSS, consisting of an electronically controlled heater/lighter device and specially designed cigarettes, puts this concept into practice. While chemical analysis and in vitro and in vivo testing of MS produced by the first generation of this system showed at least a partial reduction in yield of smoke constituents and toxic activity, it also discovered a strong increase in formaldehyde yield. To address this increase in formaldehyde, the second generation of EHCSS was developed with AMP in the cigarette paper. Chemical analysis of MS from the EHCSS with AMP showed lower yields not only of formaldehyde, but also of several other toxic tobacco smoke constituents, although there were also increases in ammonia and HMT yields (Roemer et al., 2008). The EHCSS without AMP was already characterized by a strong reduction in CO, which is considered to be a risk factor for cardiovascular diseases (U.S. Department of Health and Human Services, 2004). Incorporation of AMP resulted in an even further decrease in CO (Tables 1 and 6). Other potentially beneficial effects of AMP were seen for most of the in vitro parameters determined in cytotoxicity and mutagenicity assays (Roemer et al., 2008), which are complemented by the results of the inhalation studies presented here.

In contrast to the in vitro assays, which yield data related to specific endpoints, such as genotoxicity or cytotoxicity, the inhalation studies yield a more diverse set of data considering biological interaction in an in vivo model. To unify this diversity of measurements and create a common basis for comparison of cigarettes, we use equal effect concentration ratios (EECRs) (Terpstra et al., 2003). The EECRs in the present study are calculated from the TPM concentration of the conventional reference cigarette divided by the concentration of the test cigarette at an equal effect level. Any other smoke constituent could as well be used as a measure for the MS concentration and the basis for comparison between test atmospheres. An EECR of lower than 1 indicates that the biological activity of MS from the test cigarette is lower than that of MS from the conventional reference cigarette, notwithstanding the limits given by data variability. Likewise, an EECR of higher than 1 means that the biological activity of the EHC is greater than that of the 1R4F.

The EECRs on a TPM basis obtained in this study are slightly overestimated based on the differential composition and the resulting differential behavior of the EHCSS and conventional cigarette MS upon dilution in the exposure system as previously discussed (Terpstra et al., 2003). For example, the water concentration is generally around 10% in MS from conventional cigarettes, but around 30% in MS from the EHCSS (Stabbert et al., 2003; Roemer et al., 2004). In our experimental setup, MS is diluted between the site of smoke production (smoking machine) and the site of TPM sample collection (exposure chamber at the breathing zone of the rats, Figure 1). When the MS is diluted, the mass of the EHCSS smoke particulate matter decreases more than that of conventional cigarettes because of the partial evaporation of more abundant semi-volatile constituents, including water, glycerol, and nicotine. Thus, compared to the nominal TPM yield in undiluted MS, the TPM mass measured in the breathing zone of the rats is artificially more decreased for the EHCSS than for a conventional cigarette, which in turn leads to an overestimation of EECR values relative to the true ones, which would be based on the nominal TPM yields. This is of toxicological relevance because the rats were exposed to whole smoke, which contains an artificially increased concentration of potentially irritating constituents in the gas phase relative to TPM in MS from the EHCSS compared to that of MS from conventional cigarettes.

**FIG. 5:**
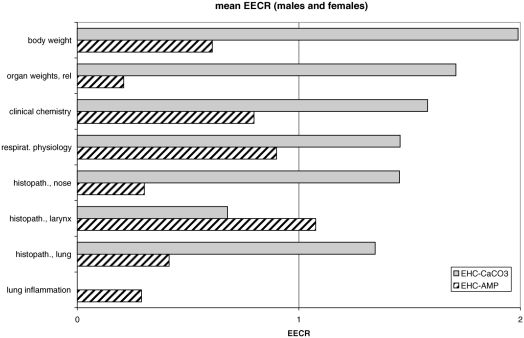
Subchronic inhalation toxicity effects of the EHC compared to the 1R4F. Summarized EECRs on a μg TPM/L basis from male and female rats for different end points combined under common toxicological perspectives. An EECR of below 1 indicates that the activity of the cigarette tested is less than that of the 1R4F.

A major point of interest in any inhalation toxicity study is the histopathological examination of the respiratory tract. Overall, MS from EHC-AMP, as indicated by the mean EECRs for histopathological findings in the nose, larynx, and lungs, showed a lower potency than that of the 1R4F (0.5 for males and 0.8 for females). In contrast, the potency of the EHC-CaCO_3_ (1.1 for males and 1.3 for females) was found to be slightly higher than that of the 1R4F. For the EHC-CaCO_3_, this compares well to an overall EECR of 1.0 obtained for the first generation of EHCSS compared to the conventional reference cigarette 1R4F (Terpstra et al., 2003). However, unlike the results obtained for the first-generation EHCSS, the EECRs obtained in the current study for the different parts of the respiratory tract do not reveal a uniform pattern for the two types of EHC. Findings in the anterior (nose) and posterior (lung) part of the respiratory tract showed considerably lower EECRs for the EHC-AMP, whereas the EECR for the larynx was somewhat higher than 1. For the EHC-CaCO_3_, the results are just the opposite, showing an EECR of slightly less than 1 for the larynx but greater than 1 for the other respiratory-tract organs (Figure 5). Laryngeal effects are mainly particulate-phase effects (Coggins et al., 1980; Friedrichs et al., 2006), although neither the TPM concentration nor the particle size distribution could explain these differences in laryngeal responses. One possible explanation might be that particle deposition is greater in the larynges of rats exposed to MS from the EHC-AMP because of the lower irritant potency of the smoke: Formaldehyde and acrolein are examples of gas-phase irritants whose concentrations were reduced in MS by the incorporation of AMP in the EHC cigarette paper. Both formaldehyde and acrolein, individually as well as in mixtures, are known to cause irritation in rats (Feron et al., 1978; Cassee et al., 1996) at the concentrations found in MS from the EHC-CaCO_3_. Despite the reductions achieved by the incorporation of AMP in the EHC cigarette paper, the acrolein concentration in MS from the EHC-AMP seen in the inhalation toxicity study is still slightly above the threshold (0.2 ppm) for acute irritation effects (Roemer et al., 1993). Rodents (like humans) can adapt their breathing patterns to accommodate exposure to irritants, and with less irritation, the inspiratory flow might be less depressed, thus leading to higher particle deposition through impaction in the larynx. This theory is supported by plethysmographic data, showing a higher average inspiratory flow in female rats and a corresponding trend for these effects in male rats in the EHC-AMP groups (Table 2). Distinctly decreased histopathological findings in the nose, which are considered to be mainly affected by the gas phase (Coggins et al., 1980; Gaworski et al., 1998; Friedrichs et al., 2006), also indicate that MS from the EHC-AMP has less irritating activity in this part of the respiratory tract.

One characteristic pathological change induced by cigarette smoke exposure in humans and experimental animals is the accumulation of inflammatory cells in the lung (Hoidal & Jeffery, 1998; Barnes, 2000). This pulmonary inflammation can lead to COPD if the normal protective and/or repair mechanisms of the lung are overwhelmed or defective (Global Initiative for Chronic Obstructive Lung Disease, 2001). Moreover, the degree of inflammation in the lung parenchyma correlates with the severity of alveolar septal damage in autopsy and surgical specimens (Eidelman et al., 1990; Finkelstein et al., 1995; Saetta, 1999).

In animal studies, the main indication of cigarette-smoke induced pulmonary inflammation is an increase in the proportion and number of neutrophils counted in lung slides or BALF (Gairola, 1986; Mordelet-Dambrine et al., 1991; Balansky et al., 1992; March et al., 1999, 2002). This increased neutrophil number has also been observed in induced sputum from human smokers, while COPD patients showed a further increase in neutrophils (Keatings et al., 1996). In the pulmonary inflammation study, a clear concentration response of BALF neutrophils was found for the 1R4F. Overall, the neutrophil numbers in BALF showed the inflammatory activity of MS from the EHC-AMP to be approximately one-third that of the 1R4F (Table 7 and Figure 5). The determination of free lung cells in BALF is, therefore, a sensitive and specific addition to the usual end points according to OECD guideline 413, when assessing the inhalation toxicity of cigarette smoke.

Changes in relative organ weights as a whole showed an EECR of below 1 for the rats exposed to MS from the EHC-AMP. A combined EECR of below 1 was also found for the EHC-AMP for clinical chemistry and reduction in body weight gain (male rats only), while the EECR for hematological activity was inconclusive. The changes in three of these four parameters, which are often indicators of systemic effects, were lower after exposure to MS from the EHC-AMP than after exposure to MS from the EHC-CaCO_3_ or the 1R4F (Figure 5).

The addition of AMP to the cigarette paper of the EHC increased the MS yield of ammonia and HMT, a condensation product of formaldehyde and ammonia (Roemer et al., 2008), and might well have increased the yield of other still unknown products. However, the results of the current in vivo studies, which utilize the broad pathological evaluation suggested by OECD guideline 413, in combination with the parallel chemical analytical and in vitro studies (Roemer et al., 2008) do not raise any toxicological concern in this regard.

The addition of AMP to the cigarette paper of the EHC reduced not only the yield and thus the MS concentration of formaldehyde, but also the yields of other smoke constituents (Roemer et al., 2008), including other aldehydes. Inhaled formaldehyde, acetaldehyde, and acrolein are absorbed and react mainly in the nose of exposed rodents (Leach et al., 1987; Wilmer et al., 1989; Morris, 1997). Consequently, the most pronounced decreases in irritative potency were observed in the nasal cavity of rats exposed to MS from the EHC with AMP in the cigarette paper compared to those exposed to MS from the EHC without AMP. However, only small percentages of these aldehydes reach the lungs upon inhalation. Nevertheless, there is a substantial decrease in the potency of EHC MS to induce bronchial goblet-cell hyperplasia with AMP incorporated in the cigarette paper. Even more pronounced is the reduction in pulmonary inflammatory potency following inhalation of MS from the EHC-AMP. This is even more noteworthy, since the already-mentioned aldehydes occur mainly in the gas phase of MS, which could not induce pulmonary inflammation in this rat inhalation model (Friedrichs et al., 2006). Thus, incorporation of AMP in the cigarette paper reduced the toxicological potency of EHC more than expected based on the intended reduction in formaldehyde yield.

In principle, different approaches can be used for comparison of cigarette smoke. According to ISO 4387 (International Organization for Standardization, 1991), analytes should be compared on a per cigarette basis. However, there is an ongoing debate as to whether other bases of comparison, such as equal TPM or equal nicotine, might be better suited to provide a link to the human situation (e.g., WHO Study Group on Tobacco Regulation, 2004). We circumvent the limitations connected with a comparison made on only one arbitrarily chosen basis by using the EECR concept (Terpstra et al., 2003). This approach not only utilizes principles well known from comparisons made on EC50 determinations, but allows for a relatively easy calculation of EECRs for a variety of other bases. Table 8 provides an overview of the histopathological findings in the respiratory tract, a key parameter of the toxicity study, calculated on a cigarette basis (cigarette/m^3^), a TPM basis (μg TPM/L), and a nicotine basis (μg nicotine/L). The comparisons on a cigarette basis reveal the lowest EECRs for both EHC types, i.e., the lowest relative potency compared to that of the conventional reference cigarette.

**TABLE 8:** Overview of equal effect concentration ratios for histopathological findings after 90 days of exposure: inhalation toxicity study

			EECRs	
			
Basis of comparison	Histopathological finding	Sex	EHC-CaCO_3_	EHC-AMP
TPM (μg/l)	Nose	M	1.43	0.20
		F	1.48	0.40
	Larynx	M	0.63	1.13
		F	0.73	1.02
	Lung	M	1.16	0
		F	1.53	0.83
Cigarette (cig./m^3^)	Nose	M	0.47	0.05
		F	0.48	0.12
	Larynx	M	0.20	0.35
		F	0.23	0.30
	Lung	M	0.38	0
		F	0.50	0.26
Nicotine (μg/l)	Nose	M	1.09	0.17
		F	1.16	0.38
	Larynx	M	0.51	0.98
		F	0.60	0.89
	Lung	M	0.91	0
		F	1.17	0.75

Mean of combined findings for each organ.

In summary, these in vivo studies show reduced toxicological activity of cigarette smoke by the addition of AMP to the paper of an electrically heated cigarette in markers considered to be linked to important risk factors for adverse health effects of cigarette smoking or to be risk factors themselves, such as CO for cardiovascular disease, clinical chemistry and organ weights for systemic toxicity, histopathological findings in the nose for irritation, and neutrophils in BALF for lung inflammation. Overall, the EECRs indicate that the inhalation toxicity on a TPM basis (i.e., per μg TPM/L) of MS from the EHC with AMP in the cigarette paper is clearly lower than that of both the conventional 1R4F and the EHC without AMP in the cigarette paper. The same trend holds true on a nicotine basis and a cigarette basis (Table 8).

Further studies are needed to clarify the relevance of these potentially beneficial effects of incorporating AMP into the cigarette paper of the EHC to the human situation.
